# Patterns of Lymphocytic Infiltrates Can Differentiate Feline Hepatic Lymphoma from Lymphocytic Portal Hepatitis

**DOI:** 10.3390/vetsci10020127

**Published:** 2023-02-07

**Authors:** Kimberley Sebastian, Rebecca C. Smedley, Alexander Bartel, Matti Kiupel

**Affiliations:** 1 Veterinary Diagnostic Laboratory, Michigan State University, Lansing, MI 48910, USA; 2 Institute for Veterinary Epidemiology and Biostatistics, Freie Universität Berlin, 14163 Berlin, Germany

**Keywords:** feline, hepatic lymphoma, portal lymphocytic hepatitis, clonality, pattern of lymphocytic infiltration, Bayesian latent class models for diagnostic test evaluation

## Abstract

**Simple Summary:**

The study aimed to distinguish between two important causes of liver disease in cats: lymphocytic inflammation and lymphoma. Both conditions are characterized by infiltration of small lymphocytes, making an accurate diagnosis difficult. Since the neoplastic cells of a lymphoma are derived from a single clone, a monoclonal population indicates a neoplastic condition in contrast to a polyclonal inflammatory condition. Clonality testing is therefore commonly used to detect lymphoma. The goal of our study was to define specific visual patterns of lymphocytic infiltrates in liver biopsies of cats which are predictive of a lymphoma or inflammation. A retrospective study was performed on 44 cats’ biopsies, and the lymphocytic infiltrates were characterized and correlated with clonality results. Four patterns of lymphocytic infiltrates were characterized: (1) tightly periportal, (2) periportal and centrilobular, (3) nodular, and (4) periportal with sinusoidal extension. Sensitivity and specificity of the lymphocytic patterns to diagnose lymphomas were analyzed against clonality results. Lymphocytic patterns 2, 3, and 4 accurately diagnosed hepatic lymphomas with a sensitivity and specificity of 82% and 77%, respectively. These four patterns of lymphocyte infiltrates provide useful diagnostic information and allow more accurate differentiation of a lymphoma from inflammation.

**Abstract:**

Hepatic lymphoma is poorly characterized in cats and differentiating between inflammation and lymphomas is often difficult. The diagnosis of hepatic lymphoma in humans relies on recognition of specific patterns of lymphocytic infiltrates and clonality testing of antigen receptors. Herein, we defined similar patterns of lymphocytic infiltrates in hepatic biopsies of cats and correlated them with clonality to determine which patterns are predictive of lymphoma. A retrospective study was performed on surgical biopsies from 44 cats. The immunophenotype was characterized using CD3 and CD20 on all 44 samples. All 44 samples were tested using PCR for T-cell receptor gamma-gene rearrangements. PCR for immunoglobulin heavy chain gene rearrangements was performed on 24 of these cats. Four patterns of lymphocytic infiltrates were characterized: (1) tightly periportal, (2) periportal and centrilobular, (3) nodular, and (4) periportal with sinusoidal extension. Other histomorphologic features (fibrosis, biliary hyperplasia, bile ductopenia, bile duct targeting, hepatic hematopoiesis, lipogranulomas, lymphonodular aggregates, other inflammatory cells) were also evaluated. The sensitivity and specificity of the lymphocytic patterns to diagnose lymphomas were determined using Bayesian Hui–Walter analysis (BLCM) against clonality results. Lymphocytic patterns 2, 3, and 4 accurately diagnosed hepatic lymphomas with a sensitivity and specificity of 82% (CI 95%: 0.65, 0.96) and 77% (CI 95%: 0.54, 1.00), respectively. None of the other microscopic features evaluated were predictive of a lymphoma or inflammation. Our study identified specific patterns of lymphocytic infiltration that differentiate feline hepatic lymphoma from inflammation while other histologic features were not associated with an accurate diagnosis.

## 1. Introduction

Feline hepatic lymphoma is poorly characterized and commonly represents a diagnostic challenge when attempting to differentiate it from lymphocytic cholangitis/periportal hepatitis [1]**.** The reasons are two-fold. Firstly, hepatic lymphocytic infiltrates in both entities are frequently small T cells, and secondly, other histomorphologic features such as fibrosis and bile duct targeting often occur with both hepatic lymphoma and lymphocytic inflammation [1]**.**

Lymphocytic cholangitis is a chronic and slowly progressive disease that can be associated with inflammatory bowel disease and pancreatitis [2,3]. Lymphocytic cholangitis has commonly been reported in 22% to 23% of cats with either feline enteropathy-associated T-cell lymphoma, small cell type (EATL type 2), or chronic enteritis [4]. Inflammatory bowel disease and EATL type 2 both occur with high frequency and similar prevalence in older cats [4,5]. In one study of 288 cats with chronic small bowel disease, 124 cats presented with EATL type 2 and 150 cats with enteritis [4]. Of the cats with EATL type 2, ten (8%) had concurrent hepatic lymphoma and twenty-eight (23%) had concurrent hepatitis. Only five (3%) cats with enteritis had concurrent hepatic lymphoma, but thirty-three (22%) had concurrent hepatitis [4]. It is important to recognize that the diagnosis of hepatitis has entirely been based on microscopic examination, and studies to investigate whether the hepatic lymphocytes represent an ascending inflammatory reaction from the damaged intestinal mucosa or spread of EATL type 2 to the liver are lacking. It is also not known whether lymphocytic cholangitis can progress to lymphoma. It has been suggested in human medicine that certain chronic inflammatory diseases may potentially increase the risk of lymphoma [6]**.** While it is feasible that the presence of chronic inflammatory conditions could also progress to lymphomas in cats, evidence for such transformation is lacking and current clinical data suggest that most cases of hepatic lymphoma and lymphocytic hepatitis represent phenotypically and clinically distinct syndromes [3].

In humans, 12% of hepatic lymphomas are of T-cell origin with most cases being peripheral T-cell lymphomas not otherwise specified (PTCL-NOS), with a CD3+/CD4+/CD8-/TCR⍺β phenotype [7,8]**,** and, as in cats, the diagnosis can be challenging. Patterns of lymphocytic infiltrate, immunophenotyping, and clonality are relied upon for an accurate diagnosis [7,8,9,10]**.** The patterns most often associated with PTCL-NOS are portal with sinusoidal extension and portal with lobular aggregates, alongside concordant immunophenotyping and TCR clonality [7]**.**

The major goal of our study was to determine patterns of lymphocytic infiltrates in liver samples from cats based on histomorphology and immunophenotype, and to determine the specificity and sensitivity of these patterns to differentiate hepatic lymphoma from lymphocytic cholangitis when using clonality testing. This approach will assist pathologists in differentiating neoplastic from inflammatory lymphocytic infiltrates in the livers of cats and potentially facilitate the prediction of clinical outcome and treatment options.

## 2. Materials and Methods

### 2.1. Study Population

A total of 44 cats were included in this retrospective study of which hepatic biopsies had been submitted to the Michigan State University Veterinary Diagnostic Laboratory between 2017 and 2022 for a feline lymphoma panel, comprising immunohistochemistry for the T and B cell markers, CD3 and CD20, respectively, followed by appropriate PCR for antigen receptor rearrangements. Cases were selected by searching the internal database using the following search terms: ‘*feline liver’*; ‘*feline hepatic’*; ‘*lymphocytic portal hepatitis*’; ‘*lymphocytic portal cholangiohepatitis*’; and ‘*hepatic lymphoma*’ and inclusion criteria included (1) sufficient sample size including at least four portal tracts [11], (2) the specimen was submitted for immunophenotyping for both CD3 and CD20, and (3) the specimen was submitted for PCR for antigen receptor rearrangements (PARR) of the T-cell receptor gamma gene (+/− PARR of the immunoglobulin heavy chain gene).

### 2.2. Histopathology

Biopsy samples of the liver were available from all cats. No additional tissue biopsies from the cats were examined for this study. All specimens were analyzed for sufficient quantity and quality for further testing. Serial sections from paraffin tissue blocks from each of the 44 cats were routinely stained with hematoxylin and eosin or further processed for immunohistochemistry for CD3 and CD20, and PARR analysis.

### 2.3. Immunohistochemistry

Serial sections were deparaffinized, rehydrated, and routinely processed for immunohistochemistry (IHC) to detect expression of CD3 and CD20 [12,13]. Briefly, immunohistochemical labeling was performed using either a polyclonal rabbit anti-human CD3 antibody (1:200; Agilent, Santa Clara, CA, USA) or a polyclonal rabbit anti-human CD20 antibody (1:200; Thermo Fisher Scientific, Waltham, MA, USA) on a Bond-Max automated system (Leica Microsystems, Bannockburn, IL, USA) with a biotin-free, polymeric peroxidase-based detection kit and DAB chromogen and hematoxylin counterstain as previously described [13]. Positive immunohistochemical controls included a normal feline lymph node to which the appropriate antisera were added. For negative controls, the primary antibodies were replaced with homologous non-immune sera.

### 2.4. DNA Preparation and PCR

DNA extraction was performed on five 10 µm-thick serial sections from the formalin-fixed paraffin-embedded tissue blocks. Tissue sections were deparaffinized with 1 mL FisherBrand CitriSolv clearing agent (Fischer Scientific, Waltham, MA, USA) and washed twice in 1 mL of 100% ethanol prior to DNA extraction using the DNeasy tissue kit (Qiagen, Valencia, CA, USA) as per the manufacturer’s recommendations. Rearrangements of T and B cell variable regions were assessed using PCR amplification to determine clonality using 5 µL of the unquantified extracted product as previously described [14]**.** The primers were designed for the immunoglobulin heavy chain variable region by Werner at al. [15] and PCR was performed as previously described by Kiupel et al. [12]. Both T and B cell PCR reactions were performed in heterotriplexes and PCR products were visualized using capillary gel electrophoresis [16].

### 2.5. Case Evaluation

**Figure 1:**
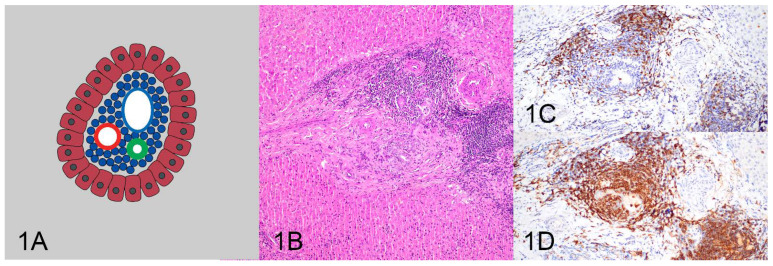
The tightly periportal pattern (**A**) consists of small lymphocytes within portal tracts (**B**) and is associated with inflammation composed of numerous T cells (**C**) and B cells forming follicles (**D**).

**Figure 2:**
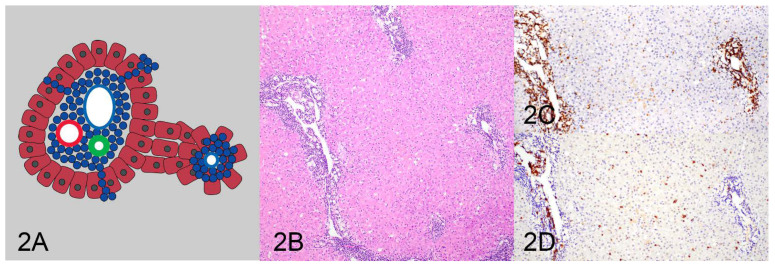
The periportal and centrilobular pattern (**A**) consists of small lymphocytes in portal tracts and surrounding at least 50% of the central veins (**B**) and is associated with T-cell lymphoma (**C**). There are few infiltrating B cells (**D**).

**Figure 3:**
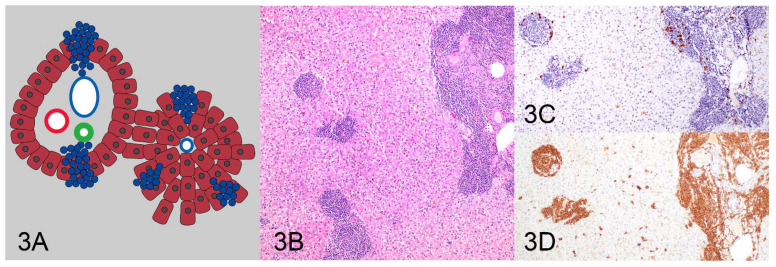
The nodular pattern (**A**) consists of randomly distributed nodular aggregates of small lymphocytes (**B**) and is mainly associated with B-cell lymphoma (**D**) and infiltrates of inflammatory T cells (**C**).

**Figure 4:**
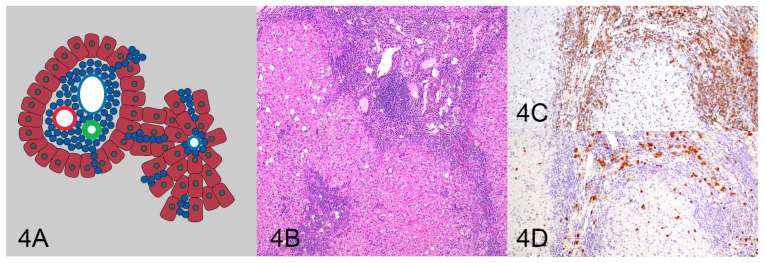
The periportal with sinusoidal extension pattern (**A**) consists of small lymphocytes that extend through the limiting plate into adjacent/randomly distributed sinusoids (**B**) and is more commonly found with T-cell lymphoma (**C**). There are few random B cells (**D**).

All HE and IHC sections were initially evaluated by one pathologist (KS) for location, distribution, and density of the lymphocytic population to determine and describe patterns of lymphocytic infiltrates. After the initial assessment, patterns were formalized as a consensus decision among three pathologists (KS, MK, RCS). Four patterns were identified as follows: (1) tightly periportal (Figure 1), (2) periportal and centrilobular (Figure 2), (3) nodular (Figure 3), and (4) periportal with sinusoidal extension (Figure 4). The tightly periportal pattern consisted of small lymphocytes present within the portal tracts only. The periportal and centrilobular pattern consisted of small lymphocytes present within portal tracts and surrounding at least 50% of the central veins in tissue sections. The nodular pattern consisted predominantly of randomly distributed nodular aggregates of small lymphocytes that may or may not occasionally involve portal tracts and central veins. The periportal with sinusoidal extension pattern consisted of small lymphocytes of which at least phalanges of 5 or more cells multifocally extended through the limiting plate and into adjacent and sometimes randomly distributed sinusoids.

In addition to the lymphocytic infiltrative patterns, a number of other parameters were evaluated that had been previously studied for their association with a diagnosis of feline hepatic lymphoma [1], including presence or absence of fibrosis, biliary hyperplasia, bile ductopenia, bile duct targeting, extramedullary hematopoiesis, lipogranulomas, lymphonodular aggregates, and other inflammatory cells.

Finally, all cases were reviewed by two blinded pathologists (MK and RCS) to determine a specific pattern for each case. If there were disagreements with regards to a final diagnosis, the cases were reviewed by all three pathologists, and a consensus diagnosis was reached for each case.

### 2.6. Statistical Analysis

All statistical analyses were performed using R version 4.2.2 (R Foundation, Vienna, Austria). To determine the diagnostic accuracy of the lymphocytic infiltration patterns as well as the other observed microscopic features in absence of a perfect reference standard (gold standard) for diagnosing lymphoma, we used a Bayesian latent class Hui–Walter model (BLCM) [14]. For dichotomization, pattern 1 was considered negative for lymphoma while patterns 2, 3, and 4 were considered positive for lymphoma. As the second test, we used the clonality results. For a 2-test 2-population model the cats were split into 2 groups by sex [17]. For both prevalences and for the sensitivities of both tests we assumed flat beta(1,1) priors. For the specificity we assumed a flat beta(1,1) prior for the lymphocytic infiltration patterns and a strong beta(1,9999) prior for the clonality results to allow for false-positive results in about 1 time in 10,000, which means the clonality results are assumed to have a nearly perfect specificity. Results are reported as median and 95% credibility intervals. As a sensitivity analysis we re-ran the model, replacing the test flat beta(1,1) priors with weakly informative beta(2,1) priors. The models were fitted with rstan (version 1.16.13) and R package bayesplot (version 1.9.0) was used for model diagnostics. Models were run using 4 chains with 2000 burn-in iterations plus 8000 sampling iterations each.

## 3. Results

The cats ranged in age from 5 years to 18 years (mean was 11 years). Twenty-two cats (50%) were neutered males, one cat (2%) was an intact male, twenty cats (46%) were neutered females, and one cat (2%) was an intact female. Thirty-four cats were domestic short hairs, there were two each of Persians and Maine coons, and one each of American shorthair and domestic longhair. Four cats were of unknown breeds.

### 3.1. Clonality

PCR for T-cell receptor gamma (TCRG) gene rearrangements was performed on all 44 cases. Thirteen cases had a clonal result for TCRG. PCR for immunoglobulin heavy chain (IgH) gene rearrangements was performed on 24 cases. As this was a retrospective study, PCR for IgH gene rearrangements only had been performed when requested by the client and material was not available to complete testing for all cases. For twenty of these cases, PCR for IgH gene rearrangements had been requested as the PCR of TCRG had only detected polyclonal gene rearrangements. Nine cases had a clonal result for IgH. One case had clonal rearrangements of both the TCRG and IgH. Therefore, a total of 21 of 44 cases were confirmed with clonal results in TCRG and/or IgH.

### 3.2. Case Evaluation

Based on data from human hepatic lymphomas [7], the tightly periportal lymphocytic pattern was expected to represent hepatitis rather than lymphoma. Eighteen of the forty-four evaluated cases had a tightly periportal lymphocytic pattern. Fifteen of these cases were polyclonal and two of the eighteen cases were clonal for IgH and one was clonal for TCRG (Table 1).

The periportal and centrilobular, the nodular, and the periportal with sinusoidal extension patterns were expected to represent lymphoma. Both, the periportal and centrilobular as well as the nodular pattern were uncommonly observed, and only detected in three and two cases, respectively. Two of the cases with a periportal and centrilobular pattern had a concurrent periportal with sinusoidal extension pattern. All five cases were clonal. The single case with only a periportal and centrilobular pattern was clonal for TCRG, while the two cases with combined periportal and centrilobular and periportal with sinusoidal extension patterns represented one case clonal for TCRG and one case clonal for IgH. The two cases with a nodular pattern also included one case clonal for TCRG and one case clonal for IgH. Both of these cases were diagnosed as B-cell lymphomas. These were the only two cases in the study diagnosed as B-cell lymphomas, all other lymphomas were T-cell lymphomas. The most commonly observed pattern was periportal with sinusoidal extension, which was detected in twenty-one cases, with thirteen of these cases being clonal (eight TCRG, five IgH), including one case being clonal for TCRG and IgH.

When applying the Baysian latent class Hui–Walter model (BLCM) to determine the accuracy of the lymphocytic patterns for diagnosing lyphoma, an 82% sensitivity and a 77% specificity for diagnosing lymphoma were detected for the periportal and centrilobular, the nodular, and the periportal with sinusoidal extension patterns versus the tightly periportal pattern (Table 2). The sensitivity of clonality was 78%. The specificity for clonality was not estimated but assumed to be nearly 100%. Sensitivity analysis with weakly informative priors showed similar results.

**Figure 5:**
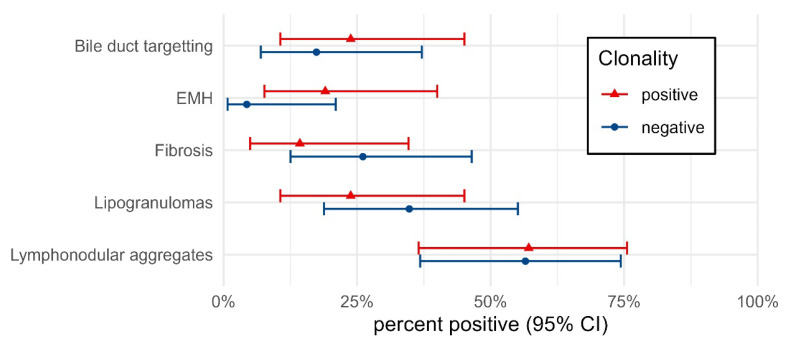
Percent of cases positive for each microscopic feature depending on clonality results. Percentages are shown with 95% confidence intervals. The small differences in percent positive between clonality groups highlight the low specificity of each feature in detecting lymphoma.

Other histomorphologic features and their prevalence in this population are shown in Table 3. None of these microscopic features accurately predicted a diagnosis of lymphoma (Figure 5).

## 4. Discussion

All four patterns of lymphocytic infiltrates described in this study differentiated lymphocytic inflammation from lymphoma in feline livers. The tightly periportal pattern was strongly associated with inflammation. The periportal and centrilobular pattern and the nodular pattern were diagnostic for lymphoma, and the periportal with sinusoidal extension pattern was moderately predictive of lymphoma. Overall, the four patterns accurately differentiated hepatic lymphoma from inflammation with a sensitivity and specificity of 82% (CI 95%: 0.65, 0.96) and 77% (CI 95%: 0.54, 1.00), respectively. Equally important, utilizing these patterns in routine diagnostics will help to standardize microscopic evaluation of lymphocytic infiltrates in feline livers and allow for consistent classification of such liver lesions and more accurate comparison of results from future studies.

While the periportal and centrilobular, and the nodular pattern were uncommon and were only observed in 11% (5/44) of cases in this study, both patterns correlated to 100% with clonality results. Furthermore, both cases with a nodular pattern were the only two B-cell lymphomas in this study population. The periportal with sinusoidal extension appeared to be moderately predictive for lymphoma. Only 62% of cases with this pattern (13/21) were clonal. The remaining eight cases with this pattern were suspected to be lymphomas but could not be definitively diagnosed as lymphomas due to negative TCRG clonality. The finding of negative clonality in these cases is potentially due to the clonal rearrangements not being amplified by the primer sets used [14]. It is important to recognize that only four of these eight cases had clonality testing for IgH performed, as material was not available for further testing. The lack of IgH clonality analysis and the technical sensitivity of the TCRG clonality testing may be partially responsible for failing to confirm clonality in these cases. This finding is echoed by the BLCM analysis, as clonality analysis misses around 20% of lymphoma cases due to its sensitivity of 78%. A recently developed TCRG assay detected clonality in 17/31 ambiguous small T-cell infiltrates in feline livers suspected to be lymphomas compared to an existing assay which detected 6/31 [18]. Interestingly, this study also identified a preferential use of TRGV7-1, TRGJ5-1, and TRGJ6-1 genes in hepatic small T-cell lymphomas, indicating these lymphomas are primary to the liver, and not metastatic from the intestine [18]. Combining the reported four patterns of lymphocytic infiltration into the liver with this newly developed TCRG assay in future studies may yield an even higher specificity than reported in this study.

For the BLCM analysis the Hui–Walter assumptions have to be met [19]. The designation of a morphological pattern and clonality testing are considered conditionally independent, since they are a visual and a genetic test, which are completely methodologically different. We think the assumption that sensitivity and specificity of both tests are independent of the populations, i.e., independent of sex, is reasonable. The difference in prevalence between populations is small (12%), but within acceptable limits [20].

Overall, primary hepatic lymphoma is uncommon in cats with a frequency of 2% (n = 185) of all lymphomas reported in one large-scale study [21]. In another study evaluating liver biopsies from 175 cats, 5% of specimens were diagnosed as lymphoma, the third most common diagnosis after hepatic lipidosis and cholangitis [22]. It is of note that these latter two studies did not provide diagnostic criteria for a diagnosis of either inflammation or lymphoma. Hepatic T-cell lymphoma appears to be much more common than hepatic B-cell lymphoma. In this study, small T-cell lymphoma was much more commonly diagnosed than diffuse large B-cell lymphoma, with 88.9% versus 11.1%, respectively. In another study, 66.6% (n = 27) of hepatic lymphomas were T-cell lymphomas [1]. This study also reported a disparity between clonality and a diagnosis of lymphoma with 17.1% of cases diagnosed with lymphocytic hepatitis having clonal rearrangements, and only 63.6% of morphologically diagnosed hepatic small T-cell lymphomas having clonal rearrangements [1]. While, as a rational for this disparity, a restricted diversity of responding inflammatory T cells in the liver or an oligoclonal TCRG repertoire of intraepithelial lymphocytes in bile ducts were suggested, the authors also acknowledged that detection of a clonal TCRG may reflect lymphomas emerging from an inflammatory condition [1].

Morphologic features previously reported to be useful for differentiation between lymphocytic cholangitis and lymphomas such as bile duct targeting, ductopenia, peribiliary fibrosis, portal B-cell aggregates, and portal lipogranulomas [1] were not shown to be differentiating features in this study and could not be used to accurately predict either a lymphoma or lymphocytic cholangitis. Our data are similar to observation of primary hepatic lymphomas in humans [10]. The discrepancy of our data to a prior study may be partially explained by the interpretation of clonality results in that previous study, where a diagnosis of inflammation was given in 17.1% of cases despite having a clonal cell population, and a lymphoma was diagnosed in 36.4% of cases without confirmation of clonality [1].

The cases chosen for this retrospective study were included due to their prior submission for immunohistochemistry and clonality testing. As such, we selected cases that were at least regarded as histologically concerning for hepatic small-cell lymphoma and may not be completely representative of all hepatic lymphocytic infiltrative diseases. Regardless, the difficulty of differentiating the two entities is highlighted by the World Small Animal Veterinary Association (WSAVA) guidelines for lymphocytic cholangitis [23], and should therefore not have significantly impacted the study overall.

As this study was conducted retrospectively, there are no data on clinical outcome. The overall prognosis of hepatic small-cell lymphoma compared to lymphocytic cholangitis in cats is unknown. Future studies should correlate histologic patterns and clonality analysis using more recently developed assays that have a higher sensitivity with clinical findings to better assess prognosis and therapeutic response. Ideally, such studies would evaluate concurrently collected intestinal and hepatic biopsies taken over multiple time points to better assess the relationship between enteropathy-associated small T-cell lymphoma and primary hepatic lymphoma as well as disease progression.

Here we describe four patterns of lymphocytic hepatic infiltrates that have an increased useability and predictability compared to identification of other histomorphologic features in differentiating lymphocytic cholangitis from hepatic small-cell lymphoma. These patterns, when utilized by pathologists, will increase concordance of diagnoses and potentially help to identify prognostic factors in the disease progression of these two entities.

## Figures and Tables

**Table 1:** Patterns of lymphocytic infiltrate and clonality. The number of cases with each pattern of lymphocytic infiltrate and the clonality results are shown.

Pattern	Clonality Negative	Clonality Positive	Total
Pattern 1: Tightly periportal	15 (83.3%)	3 (16.7%)	18 (100%)
Pattern 2: Periportal and centrilobular	0 (0%)	1 (100%)	1 (100%)
Pattern 3: Nodular	0 (0%)	2 (100%)	2 (100%)
Pattern 4: Periportal with sinusoidal extension	8 (38.1%)	13 (61.9%)	21 (100%)
Pattern 2 and 4: Periportal and centrilobular and sinusoidal extension	0 (0%)	2 (100%)	2 (100%)
Total	23 (52.3%)	21 (47.7%)	44 (100%)

**Table 2:** Bayesian latent class analysis (BLCM) results using a 2-test 2-population Hui–Walter model. Results are reported as median and 95% credibility intervals of the posterior distribution of the parameters. * Clonality specificity was assumed to be nearly perfect using a strong beta (9999, 1) prior.

Parameter	Flat Prior Model	Weakly Informative Prior Model
Pattern sensitivity	82% (65–96%)	83% (67–96%)
Pattern specificity	77% (54–100%)	79% (57–100%)
Clonality sensitivity	78% (57–100%)	79% (59–100%)
Clonality specificity *	100% (100–100%)	100% (100–100%)
Prevalence male	66% (39–90%)	65% (40–90%)
Prevalence female	54% (30–79%)	54% (31–78%)

**Table 3:** Comparison of other microscopic features against clonality. The number of each microscopic feature and the clonality results are shown in the table.

	Clonality Negative			Clonality Positive		
Histology	Cases	n	Percent (95% CI)	Cases	n	Percent (95% CI)
Bile duct targetting	23	4	17.4 (7.0–37.1)	21	5	23.8 (10.6–45.1)
EMH *	23	1	4.3 (0.8–21.0)	21	4	19.0 (7.7–40.0)
Fibrosis	23	6	26.1 (12.5–46.5)	21	3	14.3 (5.0–34.6)
Lipogranulomas	23	8	34.8 (18.8–55.1)	21	5	23.8 (10.6–45.1)
Lymphonodular aggregates	23	13	56.5 (36.8–74.4)	21	12	57.1 (36.5–75.5)

n = number of cases in each group with specific microscopic lesion. * EMH = extramedullary hematopoiesis.

## Data Availability

The data presented in this study are available on request from the corresponding author.
